# Non‐Consumptive Effects of Cannibalism Elicit a Metabolic Response in Dragonfly Larvae

**DOI:** 10.1002/ece3.70852

**Published:** 2025-02-24

**Authors:** Monika Sysiak, Jakub Baczyński, Andrzej Mikulski

**Affiliations:** ^1^ Department of Hydrobiology, Institute of Ecology, Faculty of Biology, Biological and Chemical Research Centre University of Warsaw Warsaw Poland; ^2^ Institute of Evolutionary Biology, Faculty of Biology, Biological and Chemical Research Centre University of Warsaw Warsaw Poland; ^3^ Department of Plant Biology, Miller Plant Sciences University of Georgia Athens Georgia USA

**Keywords:** cannibalism, chemical cues, dragonflies, oxygen consumption, population dynamics, respiration rate

## Abstract

Predator–prey interactions typically involve changes in metabolic rates associated with hunting, foraging, and activation/maintenance of defense mechanisms. Similar response can result from non‐consumptive effects mediated by chemical cues, such as alarm cues (indicating predation), diet cues (signaling food resources), and kairomones (indicating predator or prey availability). While the impact of interspecific chemical communication on energy expenditure is well‐studied, the role of conspecific chemical cues is less understood. This study examines non‐consumptive effects of cannibalism on metabolic rates in dragonfly larvae (*Sympetrum sanguineum*). During the respiratory experiment, larvae were exposed to low and high concentrations of conspecific kairomones and kairomones with cues from injured conspecifics to simulate different population densities and conspecific interactions. Our results showed that high concentrations of kairomones and cues from injured conspecifics significantly increased larval respiration rates in comparison with controls and low concentrations. This suggests that in an environment with constant exposure to each other's cues, larvae face ongoing readiness costs, impacting their individual fitness and population dynamics.

## Graphical abstract

Chemical cues such as kairomones and alarm cues often affect metabolic rates due to preparation for hunting, foraging, and defense. This study examines how conspecific chemical cues affect metabolic rates in dragonfly larvae (*Sympetrum sanguineum*), and finds that alarm cues and high concentrations of kairomones significantly increase larval respiratory rates. The results suggest that continuous exposure to such cues imposes an ongoing cost of readiness that affects individual fitness and population dynamics.

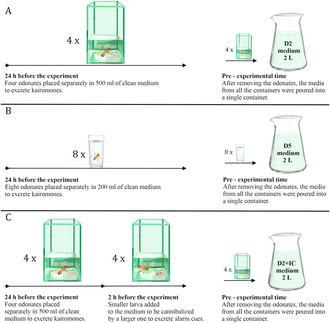

## 1 Introduction

Predator–prey interactions are energetically demanding for both predators and prey, which directly translates to a measurable increase in metabolic rate. For the predator, such costs are usually associated with active pursuit for its prey (Killen 2011; Murray et al. 2020). On the other hand, prey may boost its metabolism to allocate energy toward defensive behavior, initiating “fight or flight” response (McPeek, Grace, and Richardson 2001). A good example of such trade‐off is a burst escape mechanism in honeybees ( *Apis mellifera* ) which relies on production of costly arginine kinase enzyme (Kucharski and Maleszka 1998). However, the costs of hunting and defense are usually incurred before direct interaction takes place. Both predators and prey can use chemical communication to gauge food availability or assess predation risk (Mortensen and Richardson 2008; Zjacic and Scholz 2022). This advanced information often activates costly mechanisms that prepare individuals for hunting or defense (Riessen 1999). There are three types of such signals: alarm cues, diet cues, and kairomones. Infochemicals released by prey as a result of predator's attack can either warn other potential prey about imminent danger (alarm cues) or provide information on food availability to other predators (diet cues). Kairomones, on the other hand, are involuntarily released by both prey and predator and betray each other's presence without the necessity of direct interaction (Ferrari, Wisenden, and Chivers 2010).

The effects of chemical communication on interspecific interactions have been extensively studied (Ferrari, Wisenden, and Chivers 2010; Kaur et al. 2023; Mogali, Saidapur, and Shanbhag 2011, 2012; Riessen 1999). In contrast, our understanding of intraspecific chemical cues remains limited, likely due to difficulties in determining their direction of action. For instance, increased concentration of kairomones which typically indicates higher population density (Loose and Dawidowicz 1994), may convey information about cannibal pressure, increased competition for resources, or greater food availability. Similarly, infochemicals released by an injured individual may be interpreted as either alarm cues indicating immediate danger from conspecifics (Moir and Weissburg 2009) or as diet cues for other cannibals (Tran 2014). As in regular predation, all of the above directions of interpretation of conspecific cues by individuals should be associated with an increased metabolic rate, as a result of activation of defense mechanisms, preparation for competition for resources and/or hunting (Balderrama, de Almeida, and Núñez 1992; Pettersen et al. 2020; Robison, Chapman, and Bidwell 2018).

In typical predation scenarios, prey are only intermittently exposed to the predator's chemical cues. However, in populations where cannibalism is prevalent, individuals are constantly exposed to each other. As a result, extended periods of high population density may lead to consistently elevated metabolic rates in individuals that are prepared for frequent interactions. These long‐term costs can lead to energetic instability or anoxic stress, directly affecting the fitness of individuals (Janssens and Stoks 2013, 2020; Kolar, Boukal, and Sentis 2019; Slos and Stoks 2008). Therefore, these non‐consumptive effects of conspecifics presence may be critical for such populations in the long term which may be underestimated in the analysis of the impact of cannibalism on populations.

Odonate larvae are a well‐suited model for studies on the role of conspecific chemical cues in cannibalistic interactions. The occurrence of larval cannibalism in this group has been extensively documented (Hopper, Crowley, and Kielman 1996), and it appears to be remarkably common, particularly during early instars. Our previous investigations (Sysiak et al. 2023) have revealed that odonate larvae recognize chemical cues from conspecifics and exhibit a noticeable decrease of feeding activity in response.

We conducted a respiratory experiment in which larvae of *Sympetrum sanguineum* were exposed to (1) low and high concentrations of conspecific kairomones (representing low and high population densities, respectively) and to (2) low concentrations of conspecific kairomones with cues from injured conspecifics. Previous study demonstrated that dragonfly larvae of *Leucorrhinia dubia* increased their oxygen consumption in response to chemical cues from fish (Jiang et al. 2019). We hypothesize that *S. sanguineum* larvae exposed to conspecific chemical cues will be as follows: (a) increase oxygen consumption in response to these signals, (b) show a more intense response to high concentrations of kairomones compared to low concentrations, and (c) show an increased response to conspecific kairomones when paired with cues from injured conspecifics.

## 2 Materials and Methods

### 2.1 Experimental Animals

Dragonfly larvae *Sympetrum sanguineum* were collected in June 2021 from small pond in Warsaw (52°07′49.8″N 21°02′53.1″ E)—a natural urban lake inhabited by both fish and invertebrate predators.

Molting can significantly affect oxygen consumption in insects. To insure this process does not interfere with experiments and to accurately determine the date of their last molt, collected larvae were required to molt once under laboratory conditions (Camp, Funk, and Buchwalter 2014). During this period, the odonates were maintained at 20°C in a specially designed system as described by Sysiak et al. (2023) and fed with *Chironomidae* sp. larvae every second day. After molting, they were transferred to a thermostatic chamber in separate containers to prevent cannibalistic interactions and exposure to species‐specific signals. Low‐temperature conditions (4°C) were used to inhibit further molts, synchronizing the larvae (Shepard and Lutz 1976). To account for potential trauma‐induced behavioral changes, the experimental animals were examined for mechanical damage (e.g., leg loss) (Sesterhenn 2011) and reared in conditioned lake water filtered through a 1 μm mesh and aerated for at least two weeks.

In between molting and transfer to 4°C, larvae were photographed, and body length and head width were measured using NIS‐Elements BR 3.2 software. We also inspected the stage of wing bud development, using the number of abdominal segments covered by the wing bud as an indicator of length. Unlike other features, the wing bud in dragonfly larvae exhibits allometric growth after each molt, displaying a different growth rate compared to the rest of the organism. This discontinuity in the growth relationship between the wing bud and other parameters facilitated the assignment of collected individuals to specific size groups (Di Giovanni et al. 2000). Following Giovanni's method, scatter plots were created to examine the relationship between wing bud size and both head width and body length. These plots show the size range for each parameter associated with wing bud size (see Appendix S1). Based on this analysis, individuals were classified into three groups:

1. *Cannibals*: large individuals with head width of 4.54–4.90 mm and total body length of 11.00–12.42 mm.
2. *Prey*: small larvae with head width of 2.67–2.74 mm and total body length of 6.62–7.86 mm.
3. *Experimental animals*: intermediate sized individuals with head width of 3.48–3.75 mm and total body length of 8.24–9.82 mm. Their reaction to chemical cues was measured during experiment.

As head width constrains the maximum prey size in anisopteran larvae, larger individuals were selected to represent a genuine threat of cannibalism to experimental animals, ensuring that their kairomones are a reliable signal of potential threat.

### 2.2 Media Preparation

Before the experiment, all cannibals were acclimated to 20°C for 3 days. Cannibals were starved prior to the experiment to prevent kairomone contamination with food particles that could act as potential alarm cues. This step also increased their propensity for cannibalism. A day before the experiment cannibals were transferred to separate containers to prevent antagonistic interactions between them.

**FIGURE 1:**
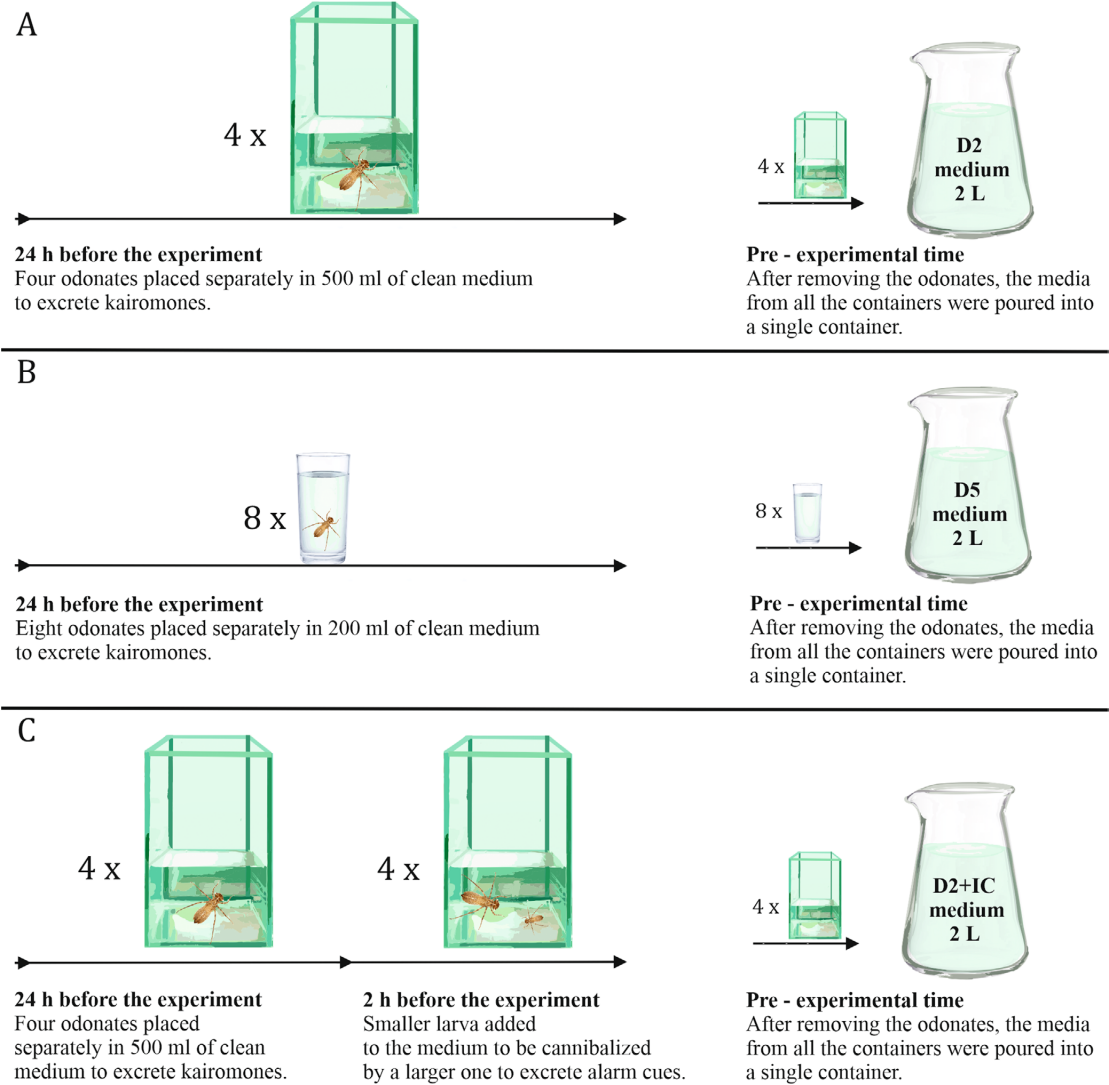
Experimental media preparation (A) D2: Kairomone from 2 *S. sanguineum* × L^−1^, (B) D5: Kairomone from 5 *S. sanguineum* × L^−1^, (C) D2 + IC: Similar to D2 but with additional cue from injured conspecifics. The arrows represent timelines of experimental media preparation. Glass vessels containing cannibals indicate low (A) and high (B) volumes, corresponding to low and high concentrations of kairomone cues, respectively. The numbers next to the vessels indicate replicates. (C) Contains an extra vessel labeled 2 h before experiment, marking the subsequent step necessary for D2 + IC medium preparation.

The experimental media were prepared as follows:

1. *C*: Control medium—lake water filtered through a 1 μm polypropylene fiber filter and then aerated for 2 weeks. Kairomone free medium.
2. *D2*: Medium with kairomones in concentration of two *S. sanguineum* × L^−1^, reflecting low population density. Each of the four cannibals were placed in 500 mL of clean medium for 24 h (Figure 1A).
3. *D5*: Medium with kairomones in concentration of five *S. sanguineum* × L^−1^, reflecting high population density. Each of the eight cannibals was placed in 200 mL of clean medium for 24 h (Figure 1B).
4. *D2* ± *IC*: Medium with cues from injured conspecifics and low concentration of kairomones mimicking low population density with additional information about interaction between conspecifics. Apart from cannibalism being allowed between odonates, the preparation of medium was identical to D2. Small larvae (defined as prey) were placed in a container with kairomone‐producing cannibals only for the last 2 h of exposition to prevent alterations to the concentration of kairomones. It was a one‐to‐one interaction. Four cannibals, each in a separate 500 mL container, were fed with one prey larvae each (Figure 1C).

Media from all replicates for each treatment were poured into a single container and filtered through a 0.2 μm antibacterial filter. The final medium was aerated for 15 min before the experiment.

The concentrations of kairomone were chosen based on field observations made during sampling. Larvae were collected from six 1 m^2^ transects, each yielding approximately 20 larvae. With an assumed water depth of 10 cm, this corresponds to a baseline density of 2 larvae per liter. A higher density of five larvae per liter—more than double the baseline—was used to amplify the effect of conspecific presence. Both concentrations are considered biologically reasonable, as odonate larvae exhibit uneven distribution in their natural habitats, and their population densities fluctuate seasonally (Bendell and McNicol 1995; Wissinger 1988b).

### 2.3 Respiratory Experiment

The entire experiment took 4 days to complete, with each of the four treatments repeated four times, once on each experimental day. Two days before the actual experiment, four experimental animals assigned to respective treatment were moved from cold (4°C) to warm environment (20°C). On the next day, experimental animals were transferred to 200 mL of conditioned and filtered (GFC) lake water and subjected to starvation to empty their digestive tracts. Lastly, 2 h before to the experiment, the water in containers was replaced, and larvae were rinsed to minimize the presence of additional organisms that could consume oxygen during measurements.

Oxygen content was measured using the UNICENSE MicroRespiratory System. Two breathing chambers (40 mL) were used per treatment: one with the experimental animal and one only with medium as a blank control to account for oxygen changes in the media. The chambers were filled, sealed, and placed in a water bath at 20°C. To minimize disturbances related to chamber filling and allow the animals to acclimatize, no additional activities were performed for 10 min after sealing. Initial oxygen measurements were taken over 30 s, and final measurements were recorded 7 h later, ensuring ~25% oxygen depletion. Larval body mass was measured non‐lethally by blotting excess water on laboratory paper before weighing each larva individually on a RADWAG XA110/2X balance. After weighing, larvae were returned to the culture container.

### 2.4 Data Analysis

We averaged each oxygen concentration from measurements taken over 30 s. The amount of oxygen in the chambers was calculated by multiplying its concentration by the chamber volume. Respiration rate was calculated by subtracting oxygen depletion in blank chambers (without animals) from changes in oxygen concentration in chambers with animals, accounting for non‐animal‐related oxygen loss. The oxygen concentration loss in the blank chambers was typically several orders of magnitude smaller than that in the animal chambers. The oxygen consumption over time (difference between initial and final measurements) was normalized to the weight of the experimental larvae, giving the oxygen consumption in millimoles per μg of animal body weight per hour.

Analyses were performed using the R programming language. The model for analysis was selected based on preliminary diagnostic assessments, including tests for skewness and kurtosis, the Shapiro–Wilk test for normality to evaluate the symmetry and peakedness of the data distribution, Levene's test for homogeneity of variance, and the Breusch–Pagan test for heteroscedasticity between groups. A generalized linear model (GLM) was used to evaluate the effect of the experimental treatments on the dependent variable, respiratory rate. The model was specified with a Gaussian distribution. Leverage and Cook's distance analyses identified one influential observation, which was subsequently removed. The model was then refitted to account for this adjustment. Diagnostic tests were performed to ensure the reliability of the re‐fitted model. Residual analysis and a quantile‐quantile (QQ) plot assessed deviations from assumptions and residual normality, with the Shapiro–Wilk test verifying the latter. Heteroscedasticity was tested again using the Breusch–Pagan test.

## 3 Results

**FIGURE 2:**
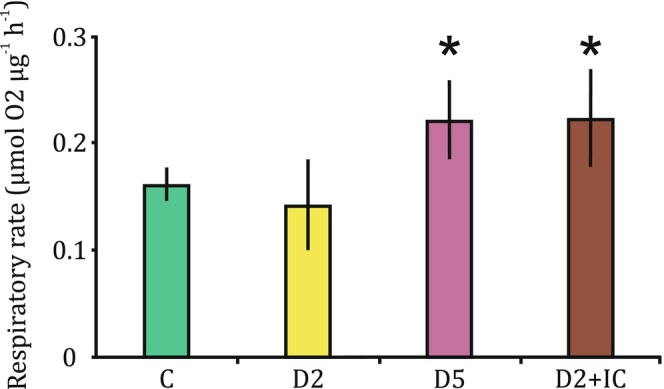
Respiratory rate (μmol O2 μg^−1^ h^−1^) of *S. sanguineum* exposed to different concentrations of kairomones (C, D2, D5) and cues from injured conspecific (D2 + IC); bars indicate standard deviation, *denotes treatments significantly different from the control treatment, C.

The Gaussian GLM analysis for respiratory rate revealed significant differences between the experimental groups compared to the control group (C). The control group had an estimated mean respiratory rate of 0.161 ± 0.018, which was highly significant (*t* = 8.988, *p* < 0.001). Compared to the control group: individuals D2 did not show a significant difference in respiratory rate (estimate = −0.019, *t* = −0.701, *p* = 0.498), D2 + IC had a significantly higher respiratory rate (estimate = 0.062 ± 0.025, *t* = 2.447, *p* = 0.032), and D5 also had a significantly higher respiratory rate (estimate = 0.060 ± 0.025, t = 2.380, *p* = 0.037) (Figure 2). The model explained variability well, reducing the residual deviance from 0.033 to 0.014. The dispersion parameter was estimated at 0.0013.

## 4 Discussion

Intraspecific interactions have been frequently described in dragonfly larvae (Hopper, Crowley, and Kielman 1996; Johansson and Crowley 2008), however, they usually focus on direct interactions rather than the non‐consumptive effects of cannibalism. The results presented herein suggest that cannibalism among odonates is prevalent enough to elicit physiological response to conspecific chemical cues that is similar as in the case of interspecific predation (Crumrine 2006; Jiang et al. 2019). In our experimental setup, odonates in the high kairomone concentration group (D5) significantly increased their respiration rate by 37.4% compared to the control group. These results are consistent with previous studies showing that cannibalism becomes more prevalent with increasing population density (Clark, Hossie, and Beresford 2021; Van Buskirk 1989). The similar response of D2 individuals to the control group (respiration rate lower only by 11.92%) suggests that the larvae were familiar with densities comparable to the low experimental concentration and perceived it as relatively safe based on their experience in their original pond. However, the significant 38.5% increase in respiration rate in the low kairomone concentration group with cues from injured conspecifics (D2 + IC) compared to the control suggests that cues from injured conspecifics can modify responses to kairomones (Pestana, Baird, and Soares 2013). In this case, the response of D2 + IC individuals closely resembles the response observed in the D5 group (Figure 2).

There are several possible explanations as to why odonate larvae exhibit increased respiration rate in response to conspecific chemical cues. As those substances can provide information about food resources, we would expect to observe higher energetic demand from larvae actively foraging for conspecifics (Killen 2011). However, libellulids, such as *Sympetrum*, are sit‐and‐wait hunters relying predominantly on sight to detect prey (Pritchard 1964). With that in mind, chemical stimuli alone are probably insufficient in triggering their searching behavior when potential prey remains well‐hidden in the vicinity. Without additional visual cue, the larvae cannot reliably assess the size of its conspecific and thus its chances of winning a fight (Ferris and Rudolf 2007). The hunting under these circumstances is not only energetically inefficient but also risky for the individual, as it becomes exposed to threat from nearby odonates and other predators. We thus suggest that the increased oxygen consumption may be associated only with readiness to attack that is ultimately triggered if conspecific appears within the range of the mask (Balderrama, de Almeida, and Núñez 1992).

Intraspecific competition is yet another possible explanation for the patterns observed in this study. Pettersen et al. (2020) have shown that in competitive environments, individuals exhibit increased metabolic rates and those with high metabolic phenotypes show greater competitive ability. This dependence, however, may be of secondary importance for odonate larvae which usually compete through interference (encounter) rather than exploitation (consumption) (Johnson et al. 1985). Moreover, in most scenarios, larvae tend to avoid each other, especially if they differ in size (Crowley et al. 1987). Additionally, as shown in our previous study (Sysiak et al. 2023), odonate larvae respond to conspecific chemical cues through increased immobility which minimizes the chance of detection by conspecifics. This strategy is more akin to a response to a predator (in this case, a cannibal) than to an intraspecific competitor. Various studies have proven that the increased oxygen consumption may be associated with activation of costly defense mechanisms (Jiang et al. 2019; Mikolajewski and Johansson 2004). For example, Murray et al. (2020), have shown that the presence of conspecifics stimulates melanization in dragonfly larvae; this process triggers a preemptive immune response and prepares for the necessity of dealing with wounds suffered from encounter with a cannibal.

The costs of anti‐cannibal defense mechanisms may have far‐reaching consequences for an individual's fitness. This is particularly true given that larvae from the same cohort reside in proximity and are constantly exposed to each other's signals (Wissinger 1988a). For example, Van Buskirk (1987) showed that coexistence with conspecifics, especially at high densities, significantly influenced the mean growth rate and reduced the survival of odonates. The fact that similar changes have been associated with non‐consumptive effects of regular predation (Lagrue, Besson, and Lecerf 2015; Sheriff et al. 2020) implies that cannibalism may be a crucial phenomenon in dragonfly larvae populations. However, as population‐level non‐consumptive predator effects are complex and difficult to predict (Sheriff et al. 2020), the understanding on how cannibalism fits into this chain of events requires further investigation.

Our results demonstrate that odonate larvae can recognize and respond to chemical cues indicating the presence of conspecifics. This is evidenced by the high metabolic costs incurred by the larvae, particularly when they receive information about high population densities and injured individuals, suggesting an imminent threat of cannibalism. In their natural environment, odonate larvae are typically exposed to each other's cues, leading to a continuous cost of readiness for antagonistic interactions with conspecifics. These non‐consumptive effects of cannibalism may represent a previously underestimated factor regulating larval dragonfly populations.

## Author Contributions

**Monika Sysiak:** conceptualization (lead), data curation (equal), formal analysis (equal), funding acquisition (lead), investigation (lead), methodology (lead), project administration (lead), supervision (lead), visualization (lead), writing – original draft (lead), writing – review and editing (lead). **Jakub Baczyński:** writing – review and editing (equal). **Andrzej Mikulski:** conceptualization (equal), data curation (equal), formal analysis (equal), methodology (supporting), writing – review and editing (equal).

## Conflicts of Interest

The author declares no conflicts of interest.

## Supporting information


Appendix S1.

## Data Availability

Data and R script with analysis are available as Supporting Information: https://figshare.com/articles/dataset/Anisoptera_respiration/26406328
