# Supplementary material for: Non‐Consumptive Effects of Cannibalism Elicit a Metabolic Response in Dragonfly Larvae
Source: Ecol Evol. 2025 Feb 24;15(2):e70852. doi: 10.1002/ece3.70852 (PMC11850442; doi:10.1002/ece3.70852)
Supplement: Supplementary file 1 — Appendix S1. [file ECE3-15-e70852-s001.pdf]

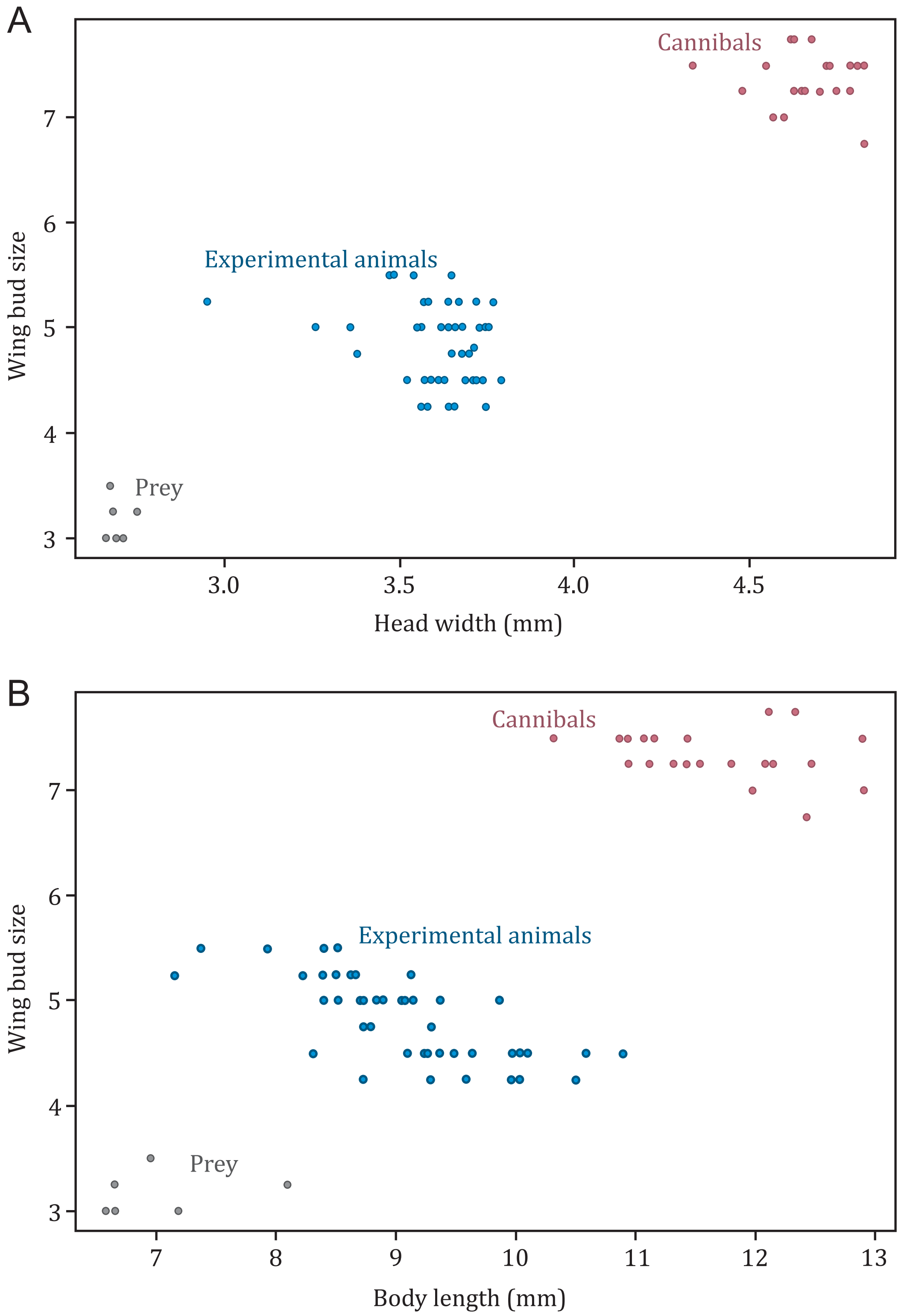

Appendix 1 (A) The relationship between wing bud size and head width of *S. sanguineum* larvae. The Y-axis represents wing bud size, measured as the number of abdominal segments covered by the wing bud, while the X-axis represents head width (mm). (B) The relationship between wing bud size and body length of *S. sanguineum* larvae. Each point represents an individual, with colors indicating experimental size groups: grey for prey of cannibals, blue for experimental animals, and red for cannibals.
